# External Validation of the FSAC Model Using On-Therapy Changes in Noninvasive Fibrosis Markers in Patients with Chronic Hepatitis B: A Multicenter Study

**DOI:** 10.3390/cancers14030711

**Published:** 2022-01-29

**Authors:** Jae Seung Lee, Hyun Woong Lee, Tae Seop Lim, In Kyung Min, Hye Won Lee, Seung Up Kim, Jun Yong Park, Do Young Kim, Sang Hoon Ahn, Beom Kyung Kim

**Affiliations:** 1 Department of Internal Medicine, Yonsei University College of Medicine, Seoul 03722, Korea; sikarue@yuhs.ac (J.S.L.); lhwdoc@yuhs.ac (H.W.L.); tslim21@yuhs.ac (T.S.L.); lorry-lee@yuhs.ac (H.W.L.); ksukorea@yuhs.ac (S.U.K.); drpjy@yuhs.ac (J.Y.P.); dyk1025@yuhs.ac (D.Y.K.); ahnsh@yuhs.ac (S.H.A.); 2 Institute of Gastroenterology, Yonsei University College of Medicine, Seoul 03722, Korea; 3 Yonsei Liver Center, Severance Hospital, Seoul 03722, Korea; 4 Department of Internal Medicine, Gangnam Severance Hospital, Yonsei University College of Medicine, Seoul 06273, Korea; 5 Department of Internal Medicine, Yongin Severance Hospital, Yonsei University College of Medicine, Yongin-si 16995, Korea; 6 Biostatistics Collaboration Unit, Department of Biomedical Systems Informatics, Yonsei University College of Medicine, Seoul 03722, Korea; iknice9@yuhs.ac

**Keywords:** carcinoma, hepatocellular, hepatitis B, chronic, antiviral agents, models, statistical, discriminant analysis, calibration, area under curve

## Abstract

**Simple Summary:**

We externally validated the recently suggested FSAC prediction model for hepatocellular carcinoma (HCC) in treatment-naïve Asian chronic hepatitis B patients starting potent antiviral therapy (AVT). The model reflects age, sex, presence of cirrhosis, and on-therapy changes in non-invasive fibrosis markers (NFMs) after 12 months of antiviral therapy, such as APRI and FIB-4. Our results highlighted better predictive performance for the FSAC model for HCC (Harrell’s c-index: 0.770) than the PAGE-B, modified PAGE-B, modified REACH-B, LSM-HCC, and CAMD models, which only use baseline parameters. A simplified version of FSAC score (i.e., FSAC (2)), including only NFMs at 12 months, also showed a high c-index value (0.763). Our retrospective study suggests that the accurate measurement of intra-hepatic fibrotic burden during adequate AVT is necessary for predicting HCC development.

**Abstract:**

Antiviral therapy (AVT) induces the regression of non-invasive fibrosis markers (NFMs) and reduces hepatocellular carcinoma (HCC) risk among chronic hepatitis B (CHB) patients. We externally validated the predictive performance of the FSAC prediction model for HCC using on-therapy NFM responses. Our multicenter study consecutively recruited treatment-naïve CHB patients (*n* = 3026; median age, 50.0 years; male predominant (61.3%); cirrhosis in 1391 (46.0%) patients) receiving potent AVTs for >18 months between 2007 and 2018. During follow-up (median 64.0 months), HCC developed in 303 (10.0%) patients. Patients with low FIB-4 or APRI levels at 12 months showed significantly lower HCC risk than those with high NFM levels at 12 months (all *p* < 0.05). Cumulative 3-, 5-, and 8-year HCC probabilities were 0.0%, 0.3% and 1.2% in the low-risk group (FSAC ≤ 2); 2.1%, 5.2%, and 11.1% in the intermediate-risk group (FSAC 3−8); and 5.2%, 15.5%, and 29.8% in the high-risk group (FSAC ≥ 9) (both *p* < 0.001 between each adjacent pair). Harrell’s c-index value for FSAC score (0.770) was higher than those for PAGE-B (0.725), modified PAGE-B (0.738), modified REACH-B (0.737), LSM-HCC (0.734), and CAMD (0.742). Our study showed that the FSAC model, which incorporates on-therapy changes in NFMs, had better predictive performance than other models using only baseline parameters.

## 1. Introduction

Chronic hepatitis B virus (HBV) infection is a major public health problem affecting approximately more than 250 million people worldwide; it remains a leading cause of hepatocellular carcinoma (HCC), especially in endemic areas such as Korea [1,2,3,4,5]. The risk of developing HCC has substantially decreased in the past several decades, stemming primarily from the use of potent oral nucleos(t)ide analogues with high genetic barriers, that is, entecavir (ETV), tenofovir disoproxil fumarate (TDF), and tenofovir alafenamide (TAF), which can effectively suppress viral replication and reduce processes of necro-inflammation and/or fibrosis [4,5,6]. Notwithstanding, since such highly active antiviral therapy cannot eradicate intra-hepatic HBV itself and the molecular mechanisms of hepato-carcinogenesis are complex [7,8,9], the regular surveillance of patients with chronic hepatitis B (CHB) is recommended to detect early stage HCC, for which treatment with a curative aim might be possible [10,11,12].

Many models have been developed to help with predicting the risk of HCC development among HBV patients with or without AVT. Since the prognostic role of baseline serum HBV-DNA levels has been substantially attenuated in the present era of potent nucleos(t)ide analogues, models established within one decade (e.g., PAGE-B, modified PAGE-B, and CAMD) have adopted the presence of baseline cirrhosis rather than virological factors [13,14,15]. Meanwhile, other HCC prediction models using fibrosis parameters assessed during long-term AVT (i.e., modified REACH-B, CAGE-B, and SAGE-B scores) have also been introduced with promising results [16,17]. Most recently, given that baseline fibrosis and/or necro-inflammation can be partially modified or regressed through long-term AVT [18,19,20], Nam et al. [21] recently suggested a novel HCC prediction model using fibrosis markers assessed at dual time points, named the Fibrosis marker response, Sex, Age, and Cirrhosis (FSAC) score. The model incorporates on-therapy changes, including a fibrosis index based on four factors (FIB-4) [22,23] and aspartate aminotransferase (AST)-to-platelet ratio index (APRI) [24] at 12 months, as well as sex, age, and cirrhosis. The model has been found to show a significantly higher predictive performance for the 10-year prediction of HCC (c-index value of 0.84) than the PAGE-B (0.77), modified PAGE-B (0.80) and REACH-B (0.67) models.

Here, we aimed to externally validate the predictive performance of the newly developed FSAC model in comparison with other risk prediction models assessed at one time-point in an independent HBV cohort treated with ETV, TDF, or TAF.

## 2. Materials and Methods

### 2.1. Study Design and Patient Follow-Up

Treatment-naïve CHB patients (age ≥ 19 years) who received AVT with ETV, TDF, or TAF between January 2007 and December 2018 at Yonsei University Severance Hospital, Gangnam Severance Hospital, and Yongin Severance Hospital, were screened for eligibility. Figure S1 depicts the flow of patient recruitment. All patients underwent transient elastography using FibroScan^®^ (EchoSens, Paris, France) at the time of AVT initiation, as described in previous reports. Cirrhosis was histologically or clinically diagnosed as follows: (1) platelet count of <150/×10^3^/μL and imaging findings suggestive of cirrhosis, including a blunted, nodular liver edge accompanied by splenomegaly (>12 cm) or (2) clinical signs of portal hypertension, such as gastroesophageal varices [25]. AVT was initiated for patients with CHB or cirrhosis according to the practice guidelines of the Korean Association for the Study of the Liver and the reimbursement guidelines of the National Health Insurance Service of Korea [26].

During follow-up, all patients underwent imaging studies (abdominal ultrasonography) and routine laboratory testing, including serum HBV-DNA, alpha-fetoprotein (AFP), and other viral markers, at 3- to 6-month intervals, as surveillance tests for HCC [5,27,28,29,30]. The primary outcome was HCC development, which was diagnosed based on histological evidence or typical radiological findings [14,31,32,33].

The study was conducted according to the guidelines of the Declaration of Helsinki and was approved by the Institutional Review Board of Yonsei University Health System, Severance Hospital (IRB No. 4-2020-0491, 22 June 2020). Patient consent was waived due to the retrospective nature of this study.

### 2.2. Non-Invasive Assessment of Fibrotic Burden and Calculation of HCC Risk Scores from Prediction Models

FSAC score was calculated based on changes in on-therapy non-invasive fibrosis markers (NFMs) at 12 months (NFMR12), as described in the literature (Table S1) [21,22,24]. The NFMR12 was classified into 4 groups: group A was defined as <3.25 and <1.45; group B as <3.25 and ≥1.45; group C as ≥3.25 and <1.45; and group D as ≥3.25 and ≥1.45 for baseline and 12-month FIB-4 indices, respectively. Group A was defined as <1.5 and <0.5, group B as ≥1.5 and <0.5, group C as <1.5 and ≥0.5, and group D as ≥1.5 and ≥0.5 for baseline and 12-month APRI, respectively [21]. Other HCC-risk prediction models, including PAGE-B, modified PAGE-B, modified REACH-B, LSM-HCC, and CAMD, were also calculated at the time of AVT initiation [13,15,34,35,36]. A simplified version of the FSAC score (FSAC [2]), which only adopted NFMs at 12 months, was also calculated (Table S1) [21].

### 2.3. Statistical Analysis

Continuous variables are expressed as medians (interquartile range [IQRs]) and were compared using Student’s *t*-tests or the Mann–Whitney U test depending on their distribution. Categorical variables are expressed as numbers (%) and were evaluated using the chi-squared test or Fisher’s exact probability test. The index date was defined as the date of AVT initiation. Patients were censored when they ended follow-up, died without HCC development, underwent liver transplantation, or developed extra-hepatic malignancy. Cox regression analysis was conducted to analyze associations between HCC development and individual risk factors and to calculate hazard ratios (HRs) with 95% confidence intervals (CI). The cumulative probability of HCC development was evaluated using the Kaplan–Meier method, and differences were assessed with the log rank test.

The predictive performances of the risk scoring models for HCC development were assessed using Harrell’s C-index, time-dependent area under the receiver operating characteristic curve (TDAUC) at 3-, 5-, and 8-years from the index date and integrated area under the receiver operating characteristic curve (iAUC). Furthermore, lower values for the Akaike information criterion (AIC) were considered reflective of a better discriminatory ability for each model. Model performance was presented graphically using calibration plots, which compared the model prediction probability with the actual probability of HCC development. Discrimination and calibration were evaluated using the bootstrap method with re-sampling 1000 times.

Statistical differences in parameters of predictive performance between FSAC and the other HCC-risk prediction models were evaluated using the bootstrap method with re-sampling 1000 times. If 95% CIs contained zero, there was deemed to be no significant difference in the parameters of predictive performance between the two models.

All statistical analyses were conducted using R package (V.4.1.1, http://cran.r-project.org/, accessed on 10 August 2021). Two-sided *p*-values < 0.05 were considered to indicate statistical significance.

## 3. Results

### 3.1. Baseline Characteristics and HCC Development

According to the enrollment criteria, a total of 3026 treatment-naïve patients were recruited. Their baseline characteristics are shown in Table 1. The median age was 50.0 (IQR 42.0–57.0) years, with a male predominance of 61.3%. In total, 1621, 1325, and 80 patients were treated with ETV, TDF, and TAF, respectively. Cirrhosis was diagnosed in 1391 (46.0%) patients, and positive hepatitis B e antigen (HBeAg) was detected in 1045 (34.5%) patients. The median values of baseline FIB-4 and APRI were 2.24 (IQR 1.30–3.75) and 0.87 (IQR 0.48–1.71), respectively. The median liver stiffness value on transient elastography was 8.3 (IQR 5.4–14.3) kPa.

During a median follow-up period of 64.0 (IQR 43.7–89.7) months, HCC developed in 303 (10.0%) patients (1.82 per 100 patient-years), and the cumulative probabilities of HCC development at 3-, 5-, and 8-years were 2.5%, 7.5%, and 15.3%, respectively. Patients who developed HCC were predominantly male (73.3% vs. 60.0%) and showed a significantly older age (median: 55.0 vs. 49.0 year); higher prevalence of cirrhosis (79.5% vs. 42.2%), diabetes mellitus (31.0% vs. 16.6%), hypertension (42.5% vs. 22.1%), and HBeAg negativity (76.2% vs. 64.3%, all *p* < 0.001); and higher median values of liver stiffness (14.3 vs. 7.7 kPa), FIB-4 (3.38 vs. 2.11), and APRI (1.09 vs. 0.84,), compared to those without HCC (all *p* < 0.001) (Table 2). Likewise, median scores for the FSAC (10 vs. 5), PAGE-B (18 vs. 13), modified PAGE-B (median: 13 vs. 11), modified REACH-B (median: 11 vs. 8), LSM-HCC (23 vs. 13), and CAMD (16 vs. 10) models were significantly higher in patients who developed HCC than those who did not (all *p* < 0.001, Table 2).

### 3.2. On-Therapy Changes in NFMs

**Figure 1:**
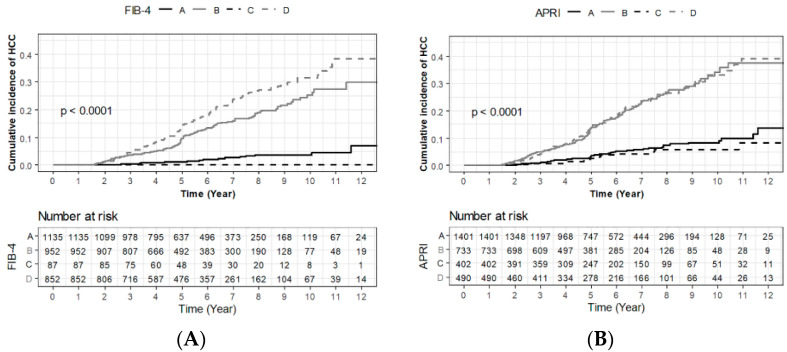
Cumulative probability of HCC development according to non-invasive fibrosis marker response groups at 12 months (groups A, B, C, and D) for FIB-4 (**A**) and APRI (**B**) among treatment-naïve CHB patients after initiating AVT. HCC, hepatocellular carcinoma; CHB, chronic hepatitis B; AVT, antiviral therapy.

According to NFMR12 with FIB-4, 1135 (37.5%), 952 (31.5%), 87 (2.9%), and 852 (28.2%) were classified into groups A, B, C, and D, respectively. Patients who developed HCC showed a higher prevalence of being classified into groups B (39.9% vs. 30.5%, respectively; *p* = 0.001) and D (52.1% vs. 25.5%, respectively; *p* < 0.001), whose FIB-4 values at 12 months were ≥1.45, compared to those without (Table S2). The cumulative probabilities of HCC development were significantly lower in groups A and C, than groups B and D (*p* < 0.001, Figure 1A).

Likewise, according to NFMR12 with APRI, 1404 (46.3%), 733 (24.2%), 402 (13.3%), and 490 (16.2%) were classified into groups A, B, C, and D, respectively. Patients who developed HCC showed a higher prevalence of being classified into groups B (42.6% vs. 22.2%, respectively; *p* < 0.001) and D (31.0% vs. 14.5%, respectively; *p* < 0.001), whose APRI values at 12 months were ≥0.5, compared to those without (Table S2). The cumulative probabilities of HCC development were significantly lower in groups A and C than groups B and D (*p* < 0.001, Figure 1B).

### 3.3. Predictive Factors of HCC Development

Univariate Cox regression analysis revealed that age, male sex, diabetes mellitus, hypertension, cirrhosis, higher liver stiffness values, lower platelet counts, higher AST, higher ALT levels, and lower serum albumin levels were significantly associated with the development of HCC (all *p* < 0.05). Subsequent multivariable analysis revealed that older age (adjusted HR 1.054, 95% CI 1.039–1.069), male sex (adjusted HR 1.959, 95% CI 1.468–2.614), presence of cirrhosis (adjusted HR 1.602, 95% CI 1.129–2.271), higher liver stiffness values (adjusted HR 1.015, 95% CI 1.005–1.025), hypertension (adjusted HR 1.370, 95% CI 1.044–1.796), and lower platelet counts (adjusted HR 0.995, 95% CI: 0.992–0.998) were independently associated with an increased risk of HCC development (all *p* < 0.05, Table S3).

### 3.4. Predictive Performance of HCC Risk Prediction Models

**Figure 2:**
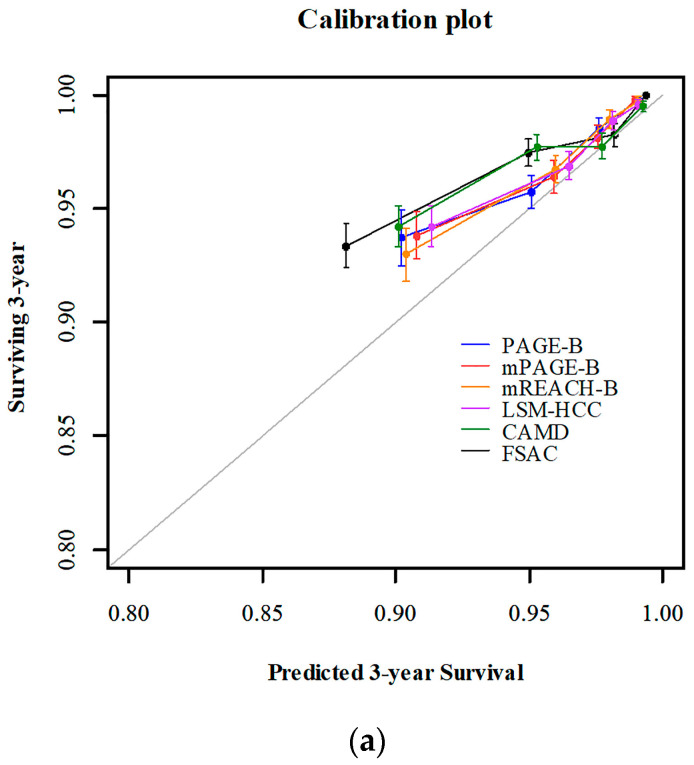

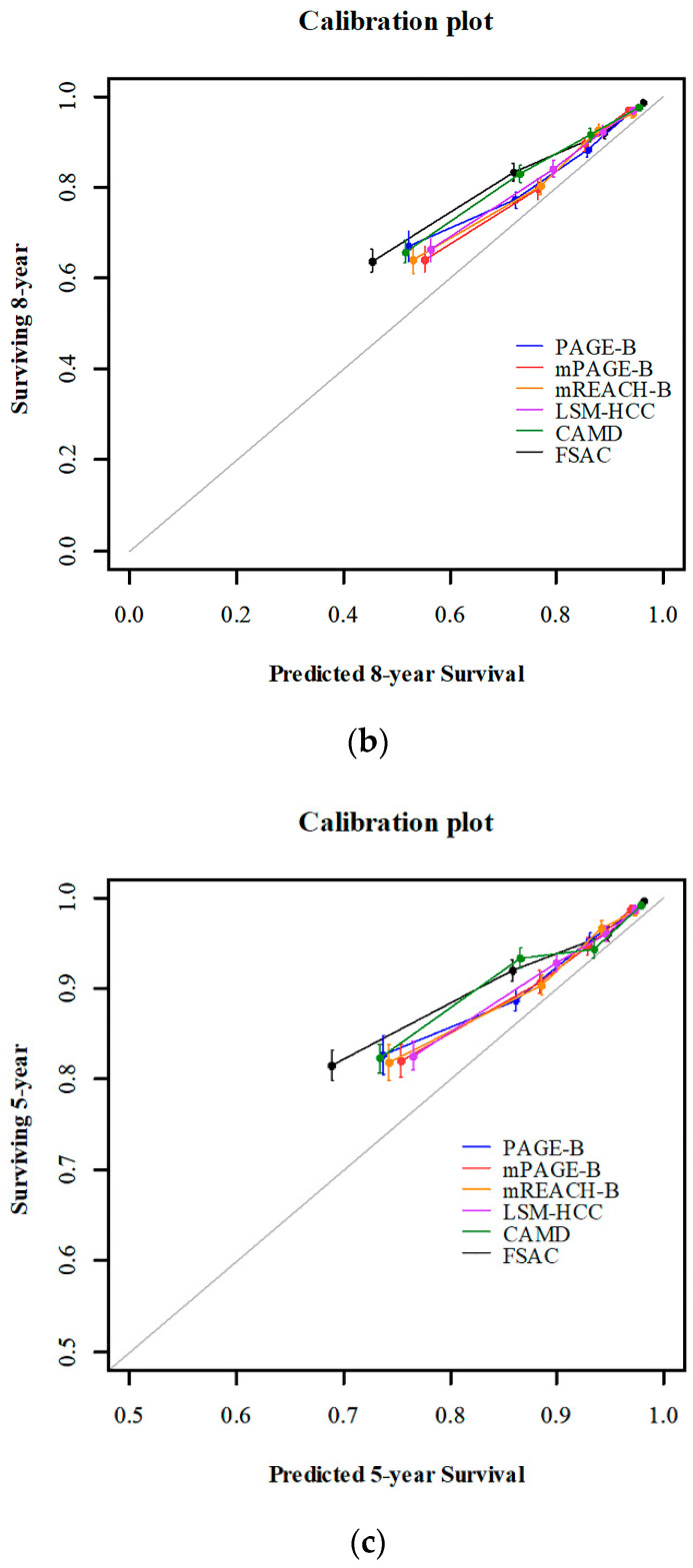
Calibration plots for FSAC and other risk prediction models for development of hepatocellular carcinoma at 3 (**a**), 5 (**b**), and 8 years (**c**) after initiating AVT. HCC, hepatocellular carcinoma; CHB, chronic hepatitis B; AVT, antiviral therapy.

Harrell’s c-index value: TDAUC values at 3-, 5-, and 8- years, and the iAUC value of the FSAC model were 0.770 (95% CI 0.745–0.794), 0.769 (95% CI 0.745–0.791), 0.768 (95% CI 0.745–0.790), 0.768 (95% CI 0.743–0.789), and 0.769 (95% CI 0.744–0.791), respectively. The calibration plots for predicting 3-, 5-, and 8-year HCC development showed that the predicted probabilities were very close to the observed incidences (Figure 2).

Harrell’s c-index values for PAGE-B, modified PAGE-B, modified REACH-B, LSM-HCC, and CAMD were 0.725 (95% CI 0.699–0.750), 0.738 (95% CI 0.712–0.764), 0.737 (95% CI 0.710–0.763), 0.734 (95% CI 0.706–0.762), and 0.742 (95% CI 0.715–0.768), respectively, whereas their iAUC values were 0.718 (95% CI 0.693–0.741), 0.722 (95% CI 0.701–0.744), 0.724 (95% CI 0.699–0.748), 0.731 (95% CI 0.705–0.755), and 0.743 (95% CI 0.717– 0.768), respectively (Table 3). The FSAC model showed significantly higher Harrell’s c-index and iAUC values than the other models (Table 4). In terms of other parameters of predictive performance (i.e., TDAUCs at 3, 5, and 8 years), the FSAC model consistently showed significantly higher performance than the PAGE-B, modified PAGE-B, modified REACH-B, LSM-HCC, and CAMD models (Table 4).

Harrell’s c-index and iAUC values of FSAC (2) model were 0.763 (95% CI 0.737–0.787) and 0.763 (95% CI 0.739–0.784), respectively (Table S4).

Subgroup analysis among patients with cirrhosis (*n* = 1391, 46.0%) showed that the Harrell’s c-index and the iAUC value of the FSAC model were 0.668 (95% CI 0.633–0.701) and 0.661 (95% CI 0.627–0.694), respectively (Table S5), and the values were higher than the other models. However, considering 95% CI, the FSAC model did not show significantly higher performance than the other models except the PAGE-B among patients with cirrhosis (Table S6).

### 3.5. HCC Risk Stratification According to FSAC Score

**Figure 3:**
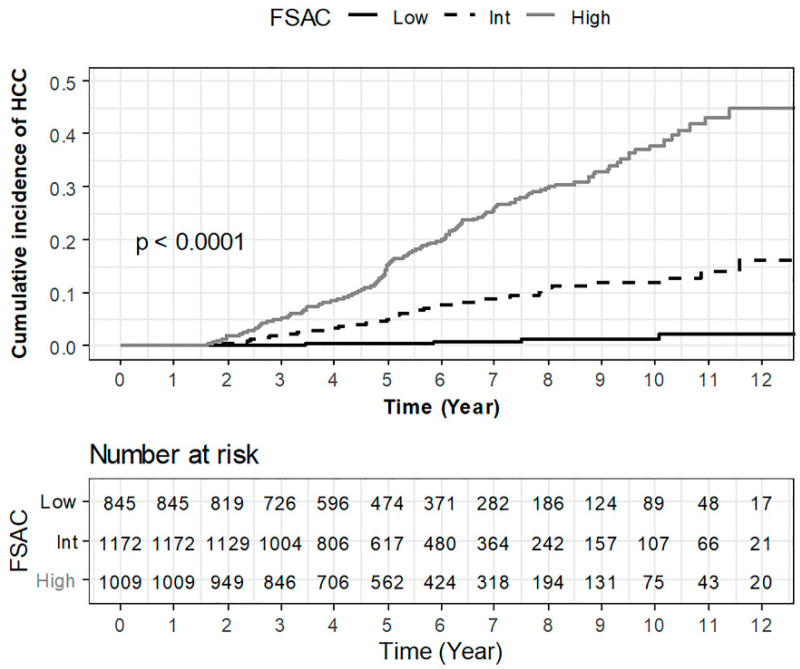
Cumulative probability of HCC development according to risk stratification by the FSAC model among treatment-naïve CHB patients after initiating AVT. HCC, hepatocellular carcinoma; CHB, chronic hepatitis B; AVT, antiviral therapy.

Using FSAC score, we stratified patients into the three risk groups: low- (*n* = 845 (27.9%): scores 0–2); intermediate- (*n* = 1172 (38.2%): scores 3–8); and high-risk groups (*n* = 1009 (33.3%): scores 9–12), according to a previous study [21]. According to such stratification, the risk of HCC development increased in a stepwise manner (*p* < 0.001, Figure 3): 6 patients in the low-risk group, 80 patients in the intermediate, and 217 patients in the high-risk group developed HCC during the follow-up period. Cumulative 3-, 5-, and 8-year HCC probabilities were 0.0% (*n* = 0), 0.3% (*n* = 2), and 1.2% (*n* = 5) in the low-risk group; 2.1% (*n* = 23), 5.2% (*n* = 48), and 11.1% (*n* = 75) in the intermediate-risk group; and 5.2% (*n* = 49), 15.5% (*n* = 126), and 29.8% (*n* = 196) in the high-risk group, respectively (both *p* < 0.001 between each adjacent pair).

## 4. Discussion

Several risk-scoring systems have been proposed to exclude HCC development within about 10 years. Most recently, Nam et al. proposed an upgraded HCC risk prediction model, that is, FSAC score, using on-therapy responses in NFMs in patients with treatment-naïve CHB. The model has been found to show superior predictive performance over other HCC-risk prediction models that incorporate only baseline factors [21]. In the present multicenter study of an independent large-scale HBV cohort, we confirmed the prognostic performance of the FSAC model to be acceptable, reliable, and superior to other HCC risk prediction models, including PAGE-B, modified PAGE-B, modified REACH-B, LSM-HCC, and CAMD, in a consistent manner.

Our study had several clinical implications. First of all, the prognostic performance of the FSAC model over other HCC-risk prediction models was reproduced. The large sample of >3000 patients with long-term follow-up enhanced the statistical reliability of the results. Moreover, a sufficient number of HCC cases (*n* = 303, 10.0%) occurred during the median follow-up period of 64.0 months, allowing for highly acceptable statistical power. Second, among the low-risk group defined according to FSAC score (*n* = 845), only 6 patients developed HCC, with an annual incidence of 0.15%. Considering that current surveillance strategies to detect early-stage HCC may be cost-effective when annual risk exceeds at least 0.2% in patients without cirrhosis and 1.5% in patients with cirrhosis, the 27.9% of individuals in the low-risk group could likely avoid biannual abdominal ultrasonography-based HCC surveillance safely. Conversely, the intermediate- and high-risk groups, accounting for the remaining 72.1% of this study population, may require more delicate surveillance, given the suboptimal diagnostic sensitivity of abdomen ultrasonography and the overall poor prognosis of HCC detected at advanced stages. Thus, in order to achieve higher detection rates of HCC at early stages and greater cost-effectiveness, further studies on how to implement individualized surveillance strategies based on optimal visit intervals and the adoption of novel diagnostic modalities using radiology and/or serum biomarkers are warranted.

Notably, we found that cumulative HCC risk tended to be more affected by NFM at 12 months itself rather than NFMR12, even though on-therapy changes in NFMs were also effective to predict HCC development. This suggests that over-estimated baseline fibrotic burden by FIB-4 and APRI, in patients with elevated AST due to necro-inflammatory activity before starting AVT, should exaggerate the degree of regressed NFM after AVT. This hypothesis is supported by the observation that the simplified version of the FSAC model (i.e., FSAC (2)) which included only NFMs assessed at 12 months, also showed a Harrell’s c-index value similar to the FSAC model (0.763, 95% CI 0.737–0.787). The observations in this study are consistent with reports of excellent predictive performance for the CAGE-B and SAGE-B models that incorporate liver stiffness values on transient elastography after 5 years of AVT.

Although the predictive performance of FSAC model among our study population was acceptable, further research using novel biomarkers that can exclude the overestimation caused by active necro-inflammation before starting AVT (e.g., three-dimensional magnetic resonance elastography) should be required in the near future [37], in order to enhance their prognostic performances for general use in routine practice. Furthermore, along with the dynamic changes in APRI or FIB-4 index during AVT, assessment of transient elastography, which proved to have higher predictive efficacy after 12 months as an easier predictive algorithm, might give useful information. Actually, when we tried to stratify the risk of HCC development by the on-treatment LS value (cutoff value: 6.4 kPa) from the subgroup with available paired TE results (*n* = 1102, 36.4%) [18], we confirmed their significant association (*p* < 0.001). Hence, further large-scale study with the serial follow-up of transient elastography during long-term AVT should be required.

Our study has several limitations. First, although liver stiffness values by transient elastography were available in all patients at the baseline, approximately two thirds of patients did not undergo transient elastography during long-term AVT, primarily because it is not reimbursed by the National Health Insurance Service in Korea. Since the predictive performances might vary depending on the sample size and HCC incidence, further studies are required in the setting of paired transient elastography tests. Second, when the HCC prediction models were assessed among a subgroup with cirrhosis, their prognostic performances were generally attenuated. This is most likely because the discriminatory power of the variables constituting the models (e.g., platelet counts, fibrosis scores, presence of cirrhosis, or reduced liver function) might become considerably attenuated in the relatively “homogenous” cirrhotic subgroup. Given that the majority of HCC rises in the setting of cirrhosis, especially among patients in Western countries, further studies concerning development of novel biomarkers should be required, allowing the optimized prognostication in a subgroup with cirrhosis.

## 5. Conclusions

In this external validation study of a large-scale cohort, the FSAC model exhibited more robust predictive performance for HCC development, compared to other HCC-risk prediction models that use only baseline characteristics. For predicting HCC development, accurate measurement of fibrotic burden during long-term AVT is necessary.

## Figures and Tables

**Table 1:** Baseline clinical characteristics of the study population.

Variables	Total (*n* = 3026)
Age (year)	50 (42, 57)
Male	1855 (61.3)
Cirrhosis	1391 (46.0)
Diabetes mellitus	547 (18.1)
Hypertension	520 (24.5)
Positive for HBeAg	1045 (34.5)
Total bilirubin (mg/dL)	0.80 (0.60, 1.10)
Serum albumin (g/dL)	4.2 (3.8, 4.4)
Platelet count (×109/L)	162 (120, 209)
Prothrombin time (INR)	1.04 (0.98, 1.12)
AST (IU/L)	50 (32, 92)
ALT (IU/L)	54 (30, 115)
Liver stiffness (kPa)	8.30 (5.40, 14.30)
FIB-4	2.24 (1.30, 3.75)
APRI	0.87 (0.48, 1.71)
PAGE-B	14 (10, 18)
Modified PAGE-B	11 (9, 13)
Modified REACH-B	8 (6, 11)
LSM-HCC	13 (6, 19)
CAMD	11 (7, 14)
FSAC	6 (2, 9)

Values are expressed as a number (%) or median (interquartile range). Abbreviations: HBeAg, hepatitis B e antigen; INR, international normalized ratio; AST, aspartate aminotransferase; ALT, alanine aminotransferase.

**Table 2:** Comparison of baseline clinical characteristics between patients with HCC and without.

Variables	No HCC (*n* = 2723)	HCC (*n* = 303)	*p* Value
Age (year)	49 (41, 56)	55 (51, 60)	<0.001
Male	1633 (60.0)	222 (73.3)	<0.001
Cirrhosis	1150 (42.2)	241 (79.5)	<0.001
Diabetes mellitus	453 (16.6)	94 (31.0)	<0.001
Hypertension	415 (22.1)	105 (42.5)	<0.001
Positive for HBeAg	973 (35.7)	72 (23.8)	<0.001
Total bilirubin (mg/dL)	0.80 (0.60, 1.10)	0.90 (0.65, 1.30)	0.053
Serum albumin (g/dL)	4.2 (3.9, 4.4)	4.0 (3.5, 4.3)	<0.001
Platelet count (×109/L)	167 (127, 213)	122 (90, 159)	<0.001
Prothrombin time (INR)	1.03 (0.97, 1.10)	1.07 (1.00, 1.16)	<0.001
AST (IU/L)	50 (32, 93)	50 (36, 86)	0.490
ALT (IU/L)	55 (30, 119)	48 (30, 87)	0.005
Liver stiffness (kPa)	7.70 (5.30, 13.10)	14.30 (9.30, 22.15)	<0.001
FIB-4	2.11 (1.25, 3.56)	3.38 (2.19, 5.76)	<0.001
APRI	0.84 (0.46, 1.68)	1.09 (0.66, 2.06)	<0.001
PAGE-B	13 (10, 16)	18 (15, 19)	<0.001
Modified PAGE-B	11 (8, 13)	13 (12, 15)	<0.001
Modified REACH-B	8 (6, 10)	11 (9, 12)	<0.001
LSM-HCC	13 (6, 19)	23 (16, 25)	<0.001
CAMD	10 (7, 14)	16 (13, 17)	<0.001
FSAC	5 (2, 9)	10 (8, 11)	<0.001

Values are expressed as a number (percentage) or median (interquartile range). Abbreviations: HCC, hepatocellular carcinoma; HBeAg, hepatitis B e antigen; AST, aspartate aminotransferase; ALT, alanine aminotransferase.

**Table 3:** Predictive performance of the FSAC and other risk-prediction models.

Scoring Systems	Harrell’s c-Index (95% CI)	3-Year TDAUC (95% CI)	5-Year TDAUC (95% CI)	8-Year TDAUC (95% CI)	iAUC (95% CI)	AIC
FSAC	0.770 (0.745, 0.794)	0.769 (0.745, 0.791)	0.768 (0.745, 0.79)	0.768 (0.743, 0.789)	0.769 (0.744, 0.791)	4156.74
PAGE-B	0.725 (0.699, 0.750)	0.724 (0.701, 0.748)	0.719 (0.697, 0.743)	0.714 (0.690, 0.737)	0.718 (0.693, 0.741)	4257.03
Modified PAGE-B	0.738 (0.712, 0.764)	0.730 (0.708, 0.753)	0.727 (0.705, 0.749)	0.719 (0.698, 0.740)	0.722 (0.701, 0.744)	4237.64
Modified REACH-B	0.737 (0.710, 0.763)	0.726 (0.701, 0.749)	0.726 (0.701, 0.749)	0.715 (0.691, 0.738)	0.724 (0.699, 0.748)	4244.98
LSM-HCC	0.734 (0.706, 0.762)	0.732 (0.705, 0.757)	0.733 (0.706, 0.757)	0.727 (0.700, 0.751)	0.731 (0.705, 0.755)	4237.24
CAMD	0.742 (0.715, 0.768)	0.744 (0.720, 0.765)	0.742 (0.718, 0.764)	0.739 (0.714, 0.763)	0.743 (0.717, 0.768)	4216.23

Abbreviations: CI, confidence interval; TDAUC, time-dependent area under the receiver operational characteristics curve; iAUC, integrated area under the receiver operational characteristics curve; AIC, Akaike information criterion.

**Table 4:** Comparison of predictive performance between the FSAC and other HCC risk-prediction models.

Comparisons	Differences of Each Parameter for Predictive Performance				
	Harrell’s c-Index (95% CI)	3-Year TDAUC (95% CI)	5-Year TDAUC (95% CI)	8-Year TDAUC (95% CI)	iAUC (95% CI)
PAGE-B vs. FSAC	0.05 (0.02, 0.07)	0.05 (0.03, 0.07)	0.05 (0.03, 0.07)	0.05 (0.03, 0.08)	0.05 (0.03, 0.07)
Modified PAGE-B vs. FSAC	0.03 (0.01, 0.05)	0.04 (0.02, 0.06)	0.04 (0.02, 0.06)	0.05 (0.03, 0.07)	0.05 (0.03, 0.07)
Modified REACH-B vs. FSAC	0.03 (0.01, 0.06)	0.04 (0.02, 0.06)	0.04 (0.02, 0.06)	0.05 (0.03, 0.08)	0.04 (0.02, 0.07)
LSM-HCC vs. FSAC	0.04 (0.01, 0.06)	0.04 (0.01, 0.06)	0.04 (0.01, 0.06)	0.04 (0.02, 0.06)	0.04 (0.01, 0.06)
CAMD vs. FSAC	0.03 (0.01, 0.05)	0.02 (0.01, 0.04)	0.03 (0.01, 0.04)	0.03 (0.01, 0.04)	0.03 (0.01, 0.04)

If 95% CI interval contains zero, there is no significant difference between two models. Abbreviations: HCC, hepatocellular carcinoma; CI, confidence interval; TDAUC, time-dependent area under the receiver operational characteristics curve; iAUC, integrated area under the receiver operational characteristics curve; LSM, liver stiffness measurement.

## Data Availability

The data presented in this study are available on request from the corresponding author. The data are not publicly available due to patient privacy concerns.

## Supplementary Materials

The following are available online at https://www.mdpi.com/article/10.3390/cancers14030711/s1, Figure S1: The Flowchart of patients’ enrollment. Table S1: Definition of components which constitute FSAC and FSAC (2), Table S2: Classification of patients according to non-invasive fibrosis marker response at 12 months after antiviral therapy for chronic hepatitis B, Table S3: Cox regression analysis for HCC development, Table S4: Comparison of predictive performance between the FSAC and FSAC (2) models, Table S5: Predictive performance of the FSAC and other risk-prediction models among patients with cirrhosis (n = 1391), Table S6: Comparison of predictive performance between the FSAC and other HCC risk-prediction models among patients with cirrhosis (n = 1391).
