# Supplementary material for: External Validation of the FSAC Model Using On-Therapy Changes in Noninvasive Fibrosis Markers in Patients with Chronic Hepatitis B: A Multicenter Study
Source: Cancers (Basel). 2022 Jan 29;14(3):711. doi: 10.3390/cancers14030711 (PMC8833581; doi:10.3390/cancers14030711)
Supplement: Supplementary file 1 [file cancers-14-00711-s001.zip › cancers-1515148-supplementary.pdf]

cancers

MDPI

Supplementary Materials

External Validation of the FSAC Model Using On-Therapy Changes in Noninvasive Fibrosis Markers in Patients with Chronic Hepatitis B: A Multicenter Study

Jae Seung Lee, Hyun Woong Lee, Tae Seop Lim, In Kyung Min, Hye Won Lee, Seung Up Kim, Jun Yong Park, Do Young Kim, Sang Hoon Ahn and Beom Kyung Kim

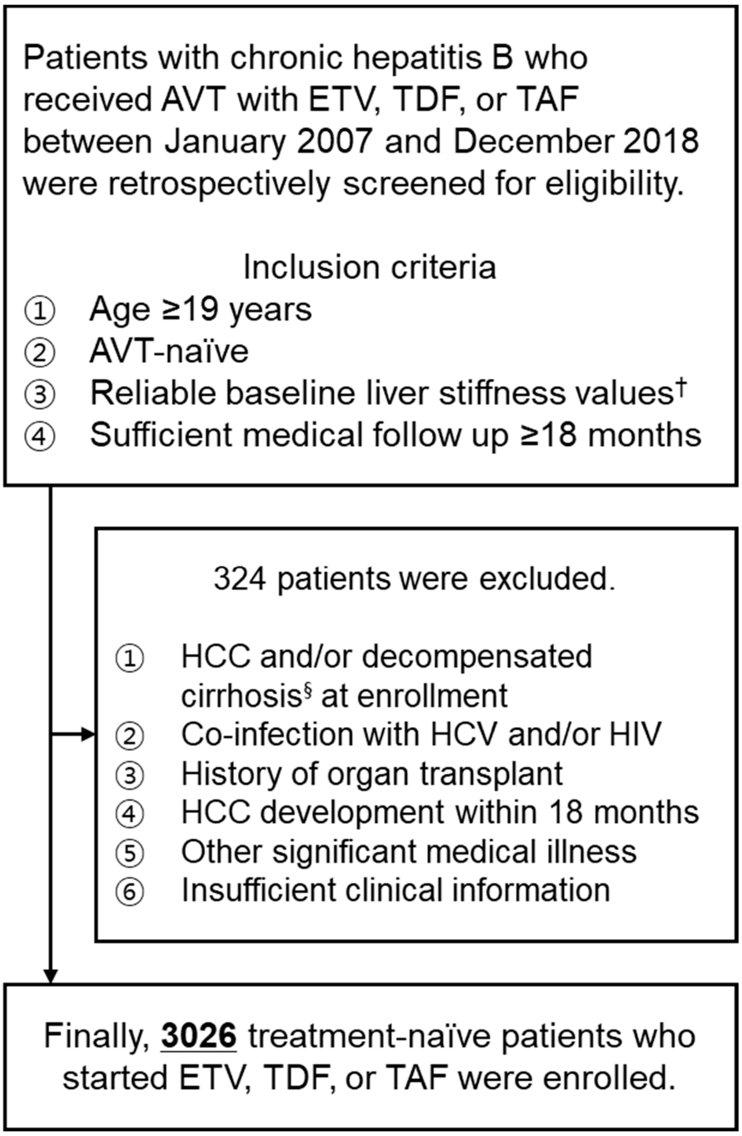

Figure S1. Flowchart of patient enrollment. § Cirrhosis was defined histologically or clinically as follows: 1) platelet count <150×10^9/L and imaging findings of a blunted, nodular liver edge accompanied by splenomegaly (>12 cm) or 2) clinical signs of portal hypertension, such as gastroesophageal varices. Decompensated cirrhosis was defined as follows: 1) ascites with diuretics use or regular paracentesis, 2) variceal bleeding, and 3) hepatic encephalopathy. Abbreviations: AVT, antiviral therapy; ETV, entecavir; TDF, tenofovir disoproxil fumarate; TAF, tenofovir alafenamide; HCC, hepatocellular carcinoma; HCV, hepatitis C virus; HIV, human immunodeficiency virus.

Table S1. Definition of the components that constitute FSAC and FSAC (2).

		By FIB-4		By APRI	
		**Baseline**	**12 months**	**Baseline**	**12 months**
NFM Response	Group A	<3.25	<1.45	<1.5	<0.5
	Group B	<3.25	≥1.45	<1.5	≥0.5
	Group C	≥3.25	<1.45	≥1.5	<0.5
	Group D	≥3.25	≥1.45	≥1.5	≥0.5

* FSAC	FIB-4 response	APRI response	Gender	Age	Cirrhosis
	Group A: 0	Group A: 0	Female: 0	<50: 0	No: 0
	Group B: 2	Group B: 2	Male: 2	≥50: 2	Yes: 3
	Group C: 0	Group C: 0			
	Group D: 3	Group D: 2			
* FSAC (2)	FIB-4 at 12 months from AVT	APRI at 12 months from AVT	Gender	Age	Cirrhosis
	<1.45: 0	<0.5: 0	Female: 0	<50: 0	No: 0
	≥1.45: 1	≥0.5: 1	Male: 1	≥50: 1	Yes: 2

* Scores represent the sum of each allocated score. Abbreviations: NFM, non-invasive fibrosis marker; AVT, antiviral therapy.

Table S2. Classification of patients according to non-invasive fibrosis marker response at 12 months after antiviral therapy for chronic hepatitis B.

**Non-Invasive Fibrosis Marker**	**Total (n = 3026)**	**No HCC (n = 2723)**	**HCC (n = 303)**	***p* value**
FIB-4 at baseline	2.24 (1.30, 3.75)	2.11 (1.25, 3.56)	3.38 (2.19, 5.76)	<0.001
FIB-4 after 12 months	1.73 (1.09, 2.77)	1.62 (1.04, 2.54)	2.87 (2.04, 4.40)	<0.001
FIB-4 response group A	1135 (37.5)	1111 (40.8)	24 (7.9)	<0.001
FIB-4 response group B	952 (31.5)	831 (30.5)	121 (39.9)	
FIB-4 response group C	87 (2.9)	87 (3.2)	0 (0.0)	
FIB-4 response group D	852 (28.2)	694 (25.5)	158 (52.1)	
APRI at baseline	0.87 (0.48, 1.71)	0.84 (0.46, 1.68)	1.09 (0.66, 2.06)	<0.001
APRI after 12 months	0.42 (0.28, 0.68)	0.39 (0.27, 0.62)	0.71 (0.49, 1.07)	<0.001
APRI response group A	1404 (46.3)	1336 (49.1)	65 (21.5)	<0.001
APRI response group B	733 (24.2)	604 (22.2)	129 (42.6)	
APRI response group C	402 (13.3)	387 (14.2)	15 (5.0)	
APRI response group D	490 (16.2)	396 (14.5)	94 (31.0)	

Abbreviations: HCC, hepatocellular carcinoma.

Table S3. Cox regression analysis for HCC development.

Variables	Univariate Analysis		Multivariate Analysis	
	p value	HR (95% CI)	*p* value	Adjusted HR (95% CI)
Male	<0.001	1.760 (1.365, 2.270)	<0.001	1.959 (1.468, 2.614)
Age (year)	<0.001	1.063 (1.052, 1.075)	<0.001	1.054 (1.039, 1.069)
TDF use (vs. ETV use)	0.885	0.982 (0.765, 1.259)	-	
TAF use (vs. ETV use)	0.899	0.880 (0.122, 6.374)	-	
Presence of cirrhosis	<0.001	4.370 (3.305, 5.777)	0.008	1.602 (1.129, 2.271)
Liver stiffness value (kPa)	<0.001	1.033 (1.026, 1.040)	0.002	1.015 (1.005, 1.025)
Diabetes mellitus	<0.001	1.999 (1.567, 2.550)	0.165	1.223 (0.920, 1.626)
Hypertension	<0.001	2.289 (1.779, 2.946)	0.023	1.370 (1.044, 1.796)
Positive for HBeAg	0.007	0.694 (0.532, 0.905)	0.623	0.922 (0.669, 1.272)
Total bilirubin (mg/dL)	0.356	1.025 (0.973, 1.080)	-	
Serum albumin (g/dL)	<0.001	0.493 (0.415, 0.585)	0.088	0.803 (0.625, 1.033)
Platelet count (×10^9/L)	<0.001	0.989 (0.987, 0.991)	<0.001	0.995 (0.992, 0.998)
AST (IU/L)	0.019	0.998 (0.997, 1.000)	0.206	0.995 (0.992, 1.001)
ALT (IU/L)	<0.001	0.997 (0.996, 0.999)	0.767	1.000 (0.997, 1.002)

Abbreviations: HCC, hepatocellular carcinoma; HR, hazard ratio; CI, confidence interval; TDF, tenofovir disoproxil fumarate; ETV, entecavir; TAF, tenofovir alafenamide; HBeAg, hepatitis B e antigen; AST, aspartate aminotransferase; ALT, alanine aminotransferase.

Table S4. Comparison of predictive performance between the FSAC and FSAC (2) models.

Scoring Systems	Harrell's C-Index (95% CI)	3-Year TDAUC (95% CI)	5-Year TDAUC (95% CI)	8-Year TDAUC (95% CI)	iAUC (95% CI)	AIC
FSAC	0.770 (0.745, 0.794)	0.769 (0.745, 0.791)	0.768 (0.745, 0.79)	0.768 (0.743, 0.789)	0.769 (0.744, 0.791)	4156.74
FSAC (2)	0.763 (0.737, 0.787)	0.764 (0.741, 0.786)	0.762 (0.74, 0.784)	0.759 (0.735, 0.782)	0.763 (0.739, 0.784)	4165.46
vs. FSAC	0.01 (0.00, 0.01)	0.01 (0.00, 0.01)	0.01 (0.00, 0.01)	0.01 (0.00, 0.01)	0.01 (0.00, 0.01)	-

If 95% CI interval contains zero, there is no significant difference between two models. Abbreviations: CI, confidence interval; TDAUC, time-dependent area under the receiver operational characteristics curve; iAUC, integrated area under the receiver operational characteristics curve; AIC, Akaike information criterion.

Table S5. Predictive performance of the FSAC and other risk-prediction models among patients with cirrhosis (n = 1391).

**Scoring systems**	Harrell's c-index (95% CI)	3-year TDAUC (95% CI)	5-year TDAUC (95% CI)	8-year TDAUC (95% CI)	iAUC (95% CI)	AIC
**FSAC**	0.668 (0.633, 0.701)	0.660 (0.625, 0.692)	0.658 (0.624, 0.69)	0.662 (0.627, 0.696)	0.661 (0.627, 0.694)	3101.55
PAGE-B	0.636 (0.598, 0.671)	0.629 (0.596, 0.664)	0.623 (0.592, 0.658)	0.621 (0.588, 0.656)	0.623 (0.590, 0.657)	3134.01
Modified PAGE-B	0.654 (0.617, 0.690)	0.646 (0.613, 0.679)	0.639 (0.608, 0.670)	0.636 (0.604, 0.665)	0.639 (0.608, 0.670)	3118.61
Modified REACH-B	0.657 (0.625, 0.688)	0.648 (0.619, 0.679)	0.648 (0.618, 0.679)	0.641 (0.611, 0.670)	0.648 (0.618, 0.678)	3114.84
LSM-HCC	0.653 (0.619, 0.688)	0.652 (0.620, 0.683)	0.653 (0.621, 0.686)	0.651 (0.618, 0.684)	0.654 (0.622, 0.688)	3111.2
CAMD	0.640 (0.602, 0.676)	0.635 (0.601, 0.669)	0.633 (0.599, 0.667)	0.634 (0.600, 0.668)	0.635 (0.600, 0.668)	3126.61

Abbreviations: CI, confidence interval; TDAUC, time-dependent area under the receiver operational characteristics curve; iAUC, integrated area under the receiver operational characteristics curve; AIC, Akaike information criterion.

Table S6. Comparison of predictive performance between the FSAC and other HCC risk-prediction models among patients with cirrhosis (n = 1391).

**Comparisons**	**Differences of each parameter for predictive performance**				
	**Harrell's c-index (95% CI)**	**3-year TDAUC (95% CI)**	**5-year TDAUC (95% CI)**	**8-year TDAUC (95% CI)**	**iAUC (95% CI)**
PAGE-B vs. FSAC	0.032 (0.005, 0.062)	0.030 (0.004, 0.057)	0.034 (0.008, 0.061)	0.041 (0.014, 0.068)	0.037 (0.011, 0.064)
Modified PAGE-B vs. FSAC	0.014 (-0.014, 0.042)	0.014 (-0.014, 0.039)	0.019 (-0.006, 0.043)	0.026 (-0.001, 0.052)	0.022 (-0.003, 0.047)
Modified REACH-B vs. FSAC	0.010 (-0.023, 0.042)	0.010 (-0.019, 0.042)	0.010 (-0.020, 0.040)	0.020 (-0.011, 0.051)	0.012 (-0.018, 0.044)
LSM-HCC vs. FSAC	0.014 (-0.019, 0.050)	0.007 (-0.024, 0.039)	0.005 (-0.026, 0.037)	0.011 (-0.020, 0.044)	0.006 (-0.026, 0.038)
CAMD vs. FSAC	0.028 (-0.003, 0.059)	0.024 (-0.003, 0.054)	0.025 (-0.003, 0.053)	0.028 (-0.003, 0.059)	0.026 (-0.004, 0.056)

If 95% CI interval contains zero, there is no significant difference between two models. Abbreviations: HCC, hepatocellular carcinoma; CI, confidence interval; TDAUC, time-dependent area under the receiver operational characteristics curve; iAUC, integrated area under the receiver operational characteristics curve; LSM, liver stiffness measurement.
